# Radiosynthesis Standardization and Preclinical Assessment of the [^68^Ga]Ga-DOTA-Ubiquicidin_29-41_: A Translational Study Targeting Differential Diagnosis of Infectious Processes

**DOI:** 10.3390/ph17010048

**Published:** 2023-12-28

**Authors:** Ana Cláudia Camargo Miranda, Leonardo Lima Fuscaldi, Jorge Mejia, Fábio Fernando Alves da Silva, Walter Miguel Turato, Fernanda Ferreira Mendonça, Solange Amorim Nogueira, Akemi Osawa, Lilian Yuri Itaya Yamaga, Luciana Malavolta, Marycel Figols de Barboza

**Affiliations:** 1 Hospital Israelita Albert Einstein, Sao Paulo 05652-900, Brazil; jorge.mecabeza@einstein.br (J.M.); solange.nogueira@einstein.br (S.A.N.); lilian.yamaga@einstein.br (L.Y.I.Y.); marycel.barboza@einstein.br (M.F.d.B.); 2 Department of Physiological Sciences, Santa Casa de Sao Paulo School of Medical Sciences, Sao Paulo 01224-001, Brazil; leonardo.fuscaldi@hotmail.com (L.L.F.); fernandaferreiramendonca@hotmail.com (F.F.M.); luciana.malavolta@gmail.com (L.M.); 3 Instituto de Pesquisas Energéticas e Nucleares, Comissão Nacional de Energia Nuclear, Sao Paulo 05508-000, Brazil; 4 School of Pharmaceutical Sciences, University of Sao Paulo, Sao Paulo 05508-000, Brazil

**Keywords:** [^68^Ga]Ga-DOTA-Ubiquicidin_29-41_, gallium-68, automated module synthesis, infectious processes, PET/CT imaging

## Abstract

Human bacterial infections significantly contribute to the increase in healthcare-related burdens. This scenario drives the study of novel techniques for the early and precise diagnosis of infectious processes. Some alternatives include Nuclear Medicine- and Molecular Imaging-based strategies. However, radiopharmaceuticals that are available for routine assessments are not specific to differentiating infectious from aseptic inflammatory processes. In this context, [^68^Ga]Ga-DOTA-Ubiquicidin_29-41_ was synthesized using an automated module and radiochemical; in vivo and in vitro studies were performed. The radiopharmaceutical remained stable in saline (up to 180 min) and in rodent serum (up to 120 min) with radiochemical purities > 99 and 95%, respectively. Partition coefficient and serum protein binding at 60 min were determined (−3.63 ± 0.17 and 44.06 ± 1.88%, respectively). Ex vivo biodistribution, as well as in vivo microPET/CT images in mice, showed rapid blood clearance with renal excretion and reduced uptake in other organs in *Staphylococcus aureus*-infected animals. Higher uptake was observed in the target as compared to the non-target tissue (*p* < 0.0001) at 60 min post administration. The presented in-human clinical case demonstrates uptake of the radiopharmaceutical by *Staphyloccocus aureus* bacteria. These results indicate the potential of [^68^Ga]Ga-DOTA-Ubiquicidin_29-41_ as a radiopharmaceutical that can be obtained in a hospital radiopharmacy for the diagnosis of infectious processes using PET/CT.

## 1. Introduction

Infectious diseases currently stand as the second leading cause of global mortality, with bacterial infections emerging as the main contributor to rising healthcare costs [1,2]. While effective disease management reduces problems related to antimicrobial resistance, the persistence of reckless prescribing practices and the prophylactic use of antibiotics still occur. A strategic approach to combat antibiotic resistance and reduce costs related to the treatment of infectious conditions is early detection and accurate diagnosis of the infectious microorganism [1,2]. Currently, only persistent infections are investigated using cultures or biopsies [3]. On the other hand, diagnosis using anatomical radiological techniques, such as computed tomography and magnetic resonance imaging, is limited by low sensitivity, since these methods may only reveal tissue alterations caused by the infectious process in more advanced stages of the disease [4].

In other ways, the images provided by Nuclear Medicine techniques, i.e., Positron Emission Tomography (PET) and Single Photon Emission Computed Tomography (SPECT), using radiopharmaceuticals, can contribute to the detection of infectious processes by scanning the patient’s whole body through non-invasive methods, differentiating them from sterile inflammation processes [2,5,6,7]. For several decades, the diagnosis of inflammatory/infectious processes has been carried out with the SPECT technique using [^111^In]In- or [^99m^Tc]Tc-exametaxime-labeled leukocytes. Although they have been classified as the gold standard, [^67^Ga]Ga-citrate and [^99m^Tc]Tc-cyprofloxacin have also been used [2,5,6,7,8,9].

Nevertheless, with the advent of PET, positron emitter-labeled molecules, such as fluorine-18 (^18^F) and gallium-68 (^68^Ga), began to be studied and developed due to the inherent advantages of this technique, such as superior spatial resolution compared to SPECT images [10,11,12]. In this sense, [^18^F]F-fluorodeoxyglucose and [^68^Ga]Ga-citrate have come to be used; however, they cannot discriminate between inflammatory and infectious processes, or distinguish them from oncological lesions [2,13,14]. In this context, studies have focused on the development of new radiolabeled molecules that comply with this purpose, such as vectors that target bacterial metabolism, bacterial cell envelopes, receptors or specific enzymes in bacteria, intracellular proteins and binding to bacterial DNA/RNA. On the other hand, several antimicrobial compounds and peptides are being radiolabeled; particularly, antimicrobial peptides, which have unique interaction characteristics with bacteria, making them promising candidates for the specific, selective and sensitive detection of infectious processes [2,15,16,17,18,19].

In this context, among the molecules of interest, can be found Ubiquicidin (UBI), an antimicrobial peptide, and its synthetic derivatives which have been suggested as possible agents for the diagnosis of infections using Nuclear Medicine [20,21,22,23]. This peptide has 59 amino acid residues, with a fragment of 29 to 41 (Thr-Gly-Arg-Ala-Lys-Arg-Arg-Met-Gln-Tyr-Asn-Arg-Arg) UBI_29-41_, proving to be important for demonstrating antimicrobial activity due to its electrostatic and hydrophobic interactions with pathogens [22,24,25,26]. Due to its high affinity, the interaction takes place on the negatively charged bacterial cell membrane, causing disturbances through different mechanisms. This characteristic, together with its non-immunogenic nature, classifies it as a suitable ligand for use in diagnostics [2].

The UBI_29-41_ fragment was previously radiolabeled with ^99m^Tc, a gamma emitter, for SPECT imaging in clinical routine [26,27]. Nowadays, ^68^Ga-labeled molecules have been developed due to some of its properties, such as being classified as a trivalent metal with a physical half-life of 67.71 min, which is consistent with the biodistribution time of low molecular weight peptides such as UBI_29-41_, and being readily available, via a generator system, for use in PET via radiopharmaceutical production in a hospital radiopharmacy [2,28].

Radiolabeling of the UBI_29-41_ fragment with ^68^Ga occurs indirectly through a bifunctional chelating agent. It is expected that the incorporation of the chelating agent into the peptide will not compromise its ability to bind selectively to bacterial cells [29]. Another point to consider is that the chelating agent can affect the radiolabeling strategy and can significantly change the parameters chosen to obtain optimal radiolabeling yields and purity. In this respect, 1,4,7-triazacyclononane-1,4,7-triacetic acid (NOTA) [26,29,30,31,32], 1,4,7,10-tetraazacyclododecane-1,4,7,10-tetraacetic acid (DOTA) [33,34,35,36,37] and 1,4,7-triazacyclonane,1-glutaric acid-4,7-acetic acid (NODAGA) [2,38,39] have been used as alternative chelating agents to be incorporated into UBI_29-41_ for radiolabeling with ^68^Ga.

In this scenario, the aim of this study was to standardize and evaluate the production of [^68^Ga]Ga-DOTA-Ubiquicidin_29-41_ in a hospital radiopharmacy unit based on radiochemical and preclinical studies (in vitro and in vivo) for the imaging detection of infectious processes, with a focus on translating it to clinical studies.

## 2. Results

### 2.1. Synthesis, Radiochemical Purity Evaluation and Stability

**Figure 1:**
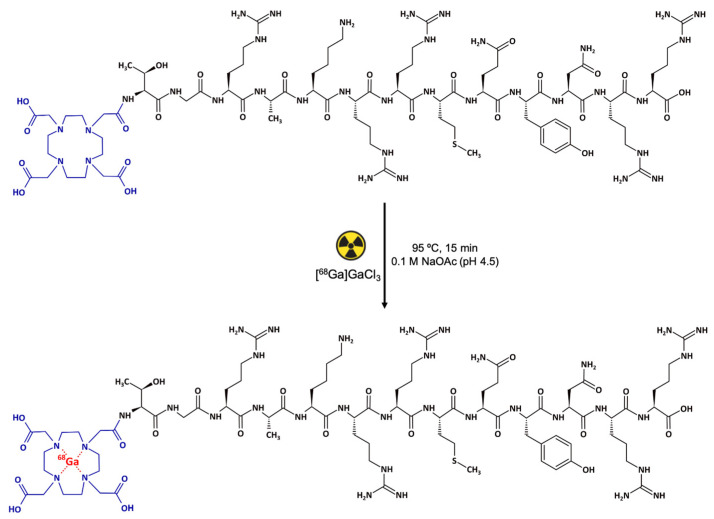
Representative scheme of the radiolabeling parameters of the DOTA-UBI_29-41_ fragment with [^68^Ga]GaCl_3_. Black: UBI_29-41_ peptide. Blue: 1,4,7,10-tetraazacyclododecane-1,4,7,10-tetraacetic acid (DOTA) bifunctional chelator. Red: [^68^Ga]Ga radionuclide.

Initially, the product was obtained via manual labeling of DOTA-UBI_29-41_ with [^68^Ga]GaCl_3_; radiochemical yield (RCY) and radiochemical purity (RCP) were determined before and after the Sep-Pak C18 cartridge-based purification step, resulting in 80.43 ± 4.10% (*n* = 4) and 98.82 ± 0.82% (*n* = 4), respectively. After this, the radiolabeling procedure was implemented in an automated synthesis module. In this case, the process consisted of the addition of 425.5 to 851.0 MBq of [^68^Ga]GaCl_3_, which was purified, in cationic filter, to 24.05 nmol of DOTA-UBI_29-41_ and diluted in 1.5 mL of 0.1 M NaOAc buffer (pH = 4.5). The reaction occurred under heating at 95 °C for 15 min (Figure 1). At the end of the procedure, 512.0 ± 3.12 MBq of [^68^Ga]Ga-DOTA-UBI_29-41_ were obtained, with a labeling yield of 81.46 ± 3.88% and an RCP, determined using solid phase extraction (SPE), of 97.80 ± 0.36%, pH = 4.5 (*n* = 14). For all the syntheses, the filter integrity test was >2.0 bar and there was no pyrogens present in the batches.

**Figure 2:**
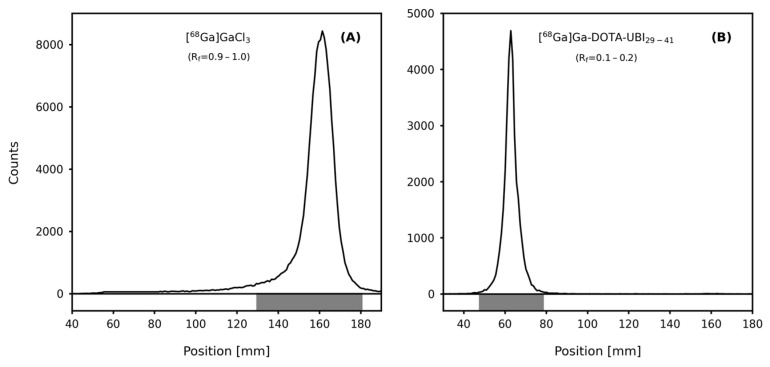
TLC chromatographic profiles of [^68^Ga]GaCl_3_ (**A**) and [^68^Ga]Ga-DOTA-UBI_29-41_ (**B**).

**Figure 3:**
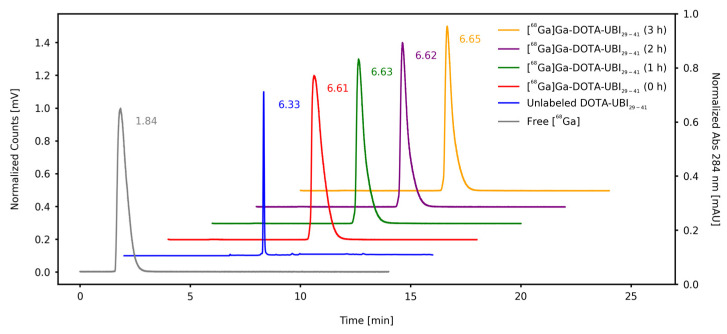
RP-HPLC chromatographic profiles of free [^68^Ga]GaCl_3_, the unlabeled DOTA-UBI_29-41_ precursor and the stability of [^68^Ga]Ga-DOTA-UBI_29-41_ at different times after radiolabeling.

RCP of the final product from the synthesis module was also determined using ascendant thin-layer chromatography (TLC) and reversed-phase High-Performance Liquid Chromatography (RP-HPLC), with values > 99% (Table 1). Using the TLC system, the radiopharmaceutical presented a retention factor (R_f_) of 0.1–0.2, while for [^68^Ga]GaCl_3_, this value was 0.9–1.0. These values indicate that this system allows the appropriate separation between the product and impurities, as shown in Figure 2. RP-HPLC chromatograms displayed that separation, with a retention time (R_t_) of 6.33 min for DOTA-UBI_29-41_, 1.84 min for free [^68^Ga]GaCl_3_ and 6.61 min for [^68^Ga]Ga-DOTA-UBI_29-41_, as shown in Figure 3.

The stability of [^68^Ga]Ga-DOTA-UBI_29-41_ in saline at room temperature was evaluated using ascendant TLC and RP-HPLC. The results are presented in Table 1.

SPE allows the quantification of the activity in the colloidal forms of the radionuclide ([^68^Ga]Ga(OH)_x_), which are retained in the filter, and of the free [^68^Ga]GaCl_3_, collected in the waste vial. Thus, both forms of impurities were considered when determining the RCP. This is why there is a small difference between the mean values obtained from the SPE (97.80 ± 0.36%) in comparison to the ascendant TLC (99.78 ± 0.06%) and RP-HPLC (99.63 ± 0.17%) techniques.

### 2.2. Partition Coefficient

Log P for [^68^Ga]Ga-DOTA-UBI_29-41_, calculated from the partition coefficient data, was –3.63 ± 0.17 (*n* = 10), confirming the hydrophilic characteristic of the radiopharmaceutical.

### 2.3. In Vitro Studies

**Figure 4:**
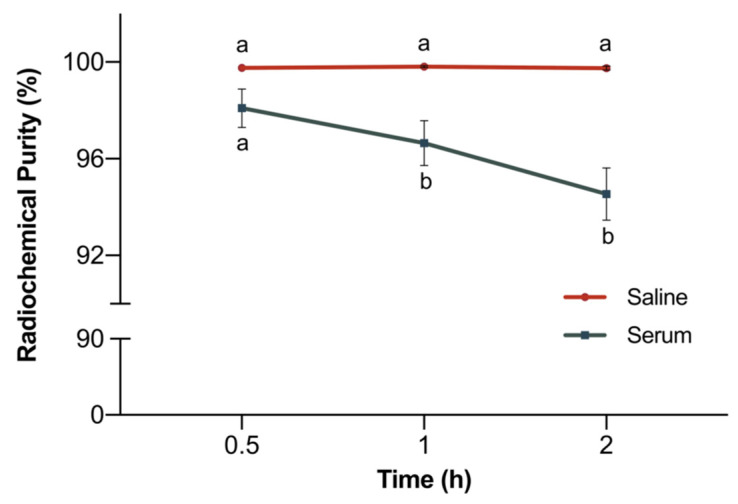
Comparison of the stability of [^68^Ga]Ga-DOTA-UBI_29-41_ in saline at room temperature and in rodent serum at 37 °C (*n* = 10). Data are expressed as mean ± SD. Different letters indicate statistically significant differences between time intervals.

The stability of the radiopharmaceutical in rodent serum at 37 °C was obtained using ascendant TLC. In Figure 4, the values obtained for 0.5, 1 and 2 h are compared with those corresponding to stability in saline, at room temperature.

The RCP of [^68^Ga]Ga-DOTA-UBI_29-41_, maintained in saline, remained above 99% throughout the time (*p* = 0.3484). In rodent serum, the RCP showed a slight reduction (*p* = 0.0005); however, it remained higher than 94%.

The binding of [^68^Ga]Ga-DOTA-UBI_29-41_ to rodent serum proteins was evaluated (*n* = 8), showing a value of 60.22 ± 2.96% at 30 min, with a reduction to 44.06 ± 1.88% at 60 min (*p* = 0.0001).

### 2.4. In Vivo Studies

**Figure 5:**
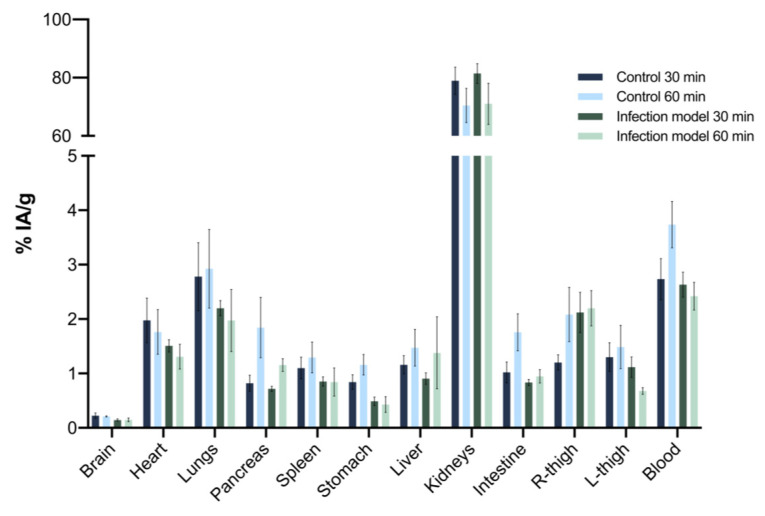
Biodistribution of [^68^Ga]Ga-DOTA-UBI_29-41_ in healthy (control) and MRSA-infected BALB/c mice, 30 and 60 min after administration of the radiopharmaceutical. Values are expressed as “mean ± SD” (*n* = 5 for each time point).

The biodistribution of [^68^Ga]Ga-DOTA-UBI_29-41_ was evaluated ex vivo in healthy and methicillin-resistant *Staphylococcus aureus* (MRSA)-infected BALB/c mice, 30 and 60 min after administration of the radiopharmaceutical (*n* = 5, for each time point). The results exhibited a fast blood depuration with urinary excretion in both healthy and infected animals. Reduced uptake was observed in other non-target organs, as shown in Figure 5.

**Figure 6:**
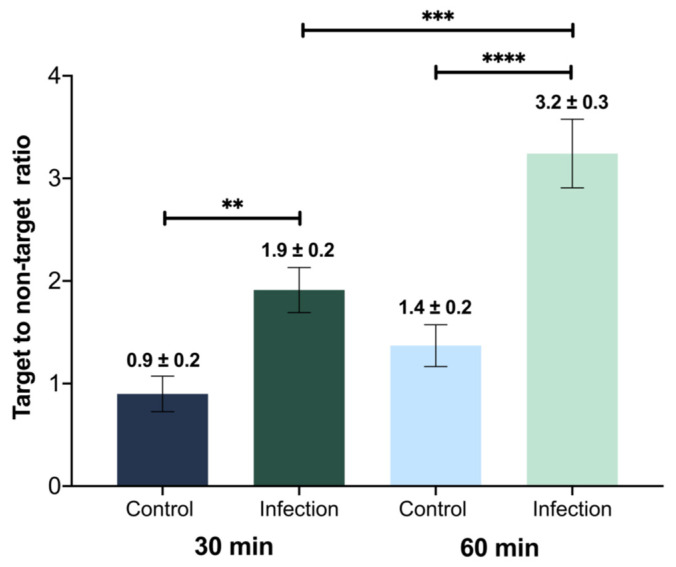
Target to non-target ratios for healthy (control) and MRSA-infected animals, at 30 and 60 min. Values are expressed as “mean ± SD” (*n* = 5). Statistically significant differences are observed. (**) *p* = 0.0012, (***) *p* = 0.0006 and (****) *p* < 0.0001. Bar colors correspond with ones in the biodistribution figure.

Target to non-target ratios were determined using the ratio of the % IA/g of the MRSA- or saline-injected to the contralateral thigh in infected and control animals, respectively. Statistically significant differences were observed between the ratios for healthy and infected animals at 30 and 60 min. A significant increase in the uptake (*p* = 0.0006) was observed in the infection site 60 min after administration of [^68^Ga]Ga-DOTA-UBI_29-41_ relative to 30 min after administration (Figure 6).

**Figure 7:**
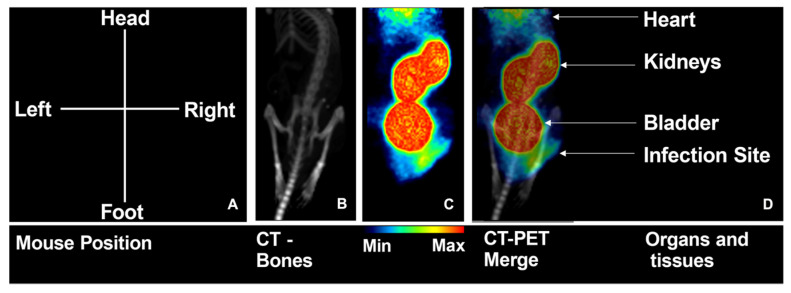
MicroPET/CT images of a MRSA-infected mouse. (**A**) Sketch of the orientation of the animal during image registration; (**B**) anatomic image (CT); (**C**) functional image (PET); and (**D**) coregistration of both PET and CT, with indication of the main uptake regions.

MicroPET/CT hybrid images of mice, obtained in dynamic mode 60 min starting at the radiopharmaceutical administration, agree with the ex vivo biodistribution data. Urinary excretion is evident from the radiopharmaceutical accumulation in the kidneys and bladder, with reduced uptake by other organs. Additionally, [^68^Ga]Ga-DOTA-UBI_29-41_ uptake was observed at the infection locus in the right thigh, which is more intense than the uptake in the contralateral thigh, as shown in Figure 7.

### 2.5. Clinical Case

**Figure 8:**
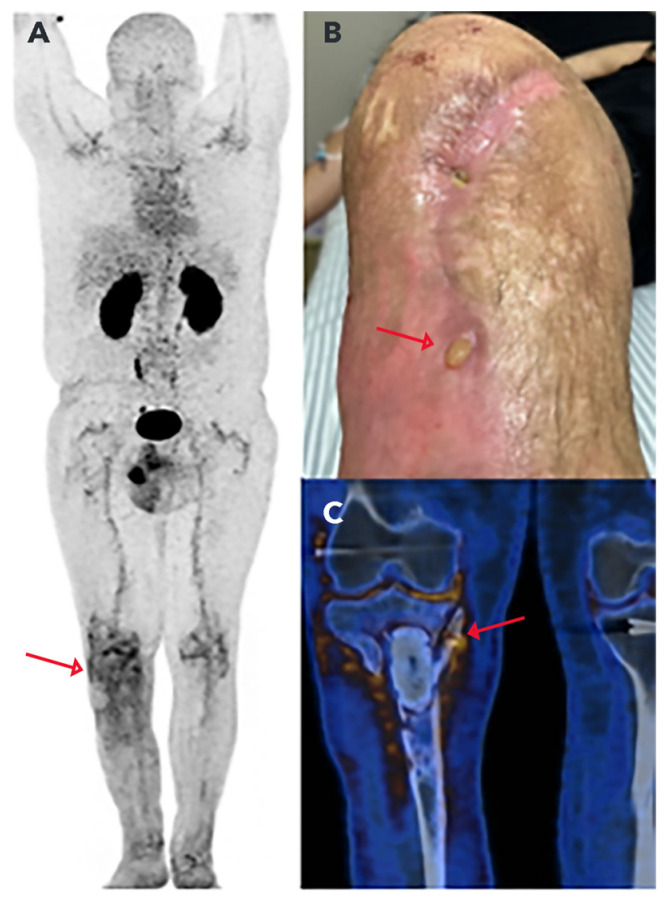
(**A**) Biodistribution of [^68^Ga]Ga-DOTA-UBI_29-41_ in the whole-body PET/CT image in maximum intensity projection (MIP). The arrow indicates the increased uptake of the radiopharmaceutical in the right knee. (**B**) The arrow indicates the lesion with purulent secretion in the proximal portion of the right leg. (**C**) Fusion of the PET/CT images, where the arrow highlights the uptake of the radiopharmaceutical in the right proximal tibia.

PET/CT images (Figure 8) showed the hydrophilic character of [^68^Ga]Ga-DOTA-UBI_29-41_, a high concentration of the radiopharmaceutical in the urinary tract, especially in the kidneys and bladder due to urinary excretion, and a low uptake in soft tissues. Detailed analysis of the proximal portion of the right tibia revealed the abnormal uptake of [^68^Ga]Ga-DOTA-UBI_29-41_ (SUV_max_ = 2.93) in soft tissues of the tibial metaphyseal region and edges of the pseudoarthrosis cavity, and trabecular irregularities in the proximal diaphysis of the tibia, in a 4.5 cm segment near the end of the cavity, with slight uptake of the radiopharmaceutical (SUV_max_ = 1.5) and minimal linear uptake in bone trabecular defects in the upper tibial plateau (SUV_max_ = 1.5). The degree of radiopharmaceutical uptake in the affected areas was higher than the physiological concentration in the liver and heart. These PET/CT findings were considered suspicious of osteomyelitis. The patient underwent a surgery, and the culture test of the intramedullary content and deep soft tissue of right tibia confirmed the presence of *Staphylococcus aureus* bacteria.

## 3. Discussion

Despite advances in the development of radiolabeled molecules and different diagnostic strategies, there is still no gold standard radiopharmaceutical available for infectious imaging diagnosis, which would help in the differential identification of pathophysiological and/or biochemical alterations associated with the infectious disease. In clinical practice, the radiopharmaceuticals [^111^In]In- or [^99m^Tc]Tc-exametaxime-labeled in vitro leukocytes, [^99m^Tc]Tc-cyprofloxacin, [^67,68^Ga]Ga-citrate and [^18^F]F-fluorodeoxyglucose have been used, but they are non-specific compounds for infection identification. The literature reveals scientific efforts to discover both bacteria-specific and -sensitive molecules [2,3,4,5,6,7,8,9]. In addition, the ideal radiopharmaceutical should be non-toxic, widely available through an easy and rapid synthesis process and should present the optimal biochemical properties, such as moderate lipophilicity, low binding to plasma proteins, metabolic stability, adequate pharmacokinetics with rapid blood clearance [40]. In this sense, antimicrobial peptides are being studied because of their suitable properties for this purpose, especially Ubiquicidin_29-41_.

Antimicrobial peptide UBI_29-41_ was previously radiolabeled with [^68^Ga]GaCl_3_ from different types of generators with several chelating agents and radiosynthesis methods (manual and automated), employing distinct labeling conditions [2].

The protocol for the synthesis of a radiopharmaceutical in a hospital radiopharmacy must be standardized, taking into account the conditions chosen for the involved processes and training personal, as well as the equipment and infrastructure present on site. In addition, the final product must be pure and sterile [2]. Thus, in this study, the radiolabeling conditions for DOTA-UBI_29-41_ used the cationic filter to purify the [^68^Ga]GaCl_3_ eluate, obtained from a generator and produced under the Good Manufacturing Practices (GMP) available for human use. Percentages of RCP in the final product of each synthesis were satisfactory and greater than 99% using an automated module. Determination of RCP using SPE stood out for being able to distinguish two impurity species, free [^68^Ga]GaCl_3_ and [^68^Ga]Ga(OH)_x_; however, the latter could not be determined using the protocols chosen for the ascendant TLC and RP-HPLC techniques. In addition, it is important to emphasize that SPE is a quick technique to perform and is compatible with the production of radiopharmaceuticals that contain a radionuclide which has a short half-life. The total time to produce the radiopharmaceutical, to carry out the relevant quality controls and to dispense the final product to the patient was optimized at 30 min, which is considered adequate for its incorporation into the routine of a hospital’s radiopharmacy unit.

[^68^Ga]Ga-DOTA-UBI_29-41_ remained stable in saline and rodent serum (RCP > 94%) during the studied period (up to 2 h). Results reported in the literature show the stability of the product in human plasma after up to 4 h of incubation (RCP > 90%) [36,37]. Sriwiang et al. assessed the binding of [^68^Ga]Ga-UBI_29-41_ to human’s plasma proteins and obtained values between 50 and 60% [36]. In the present study, although the SPB assay was carried out on rodent serum, the results are in accordance with Sriwiang et al.’s findings with 60% binding in 30 min.

To the best of our knowledge, there are no results regarding the partition coefficient of [^68^Ga]Ga-DOTA-UBI_29-41_. The data obtained in this work reveal that this compound is hydrophilic (Log P = −3.63), with similar values to the [^68^Ga]Ga-NOTA-UBI and [^68^Ga]Ga-NODAGA-UBI [31,38,41].

Ex vivo biodistribution and in vivo imaging studies on BALB/c mice confirm the hydrophilic nature of [^68^Ga]Ga-DOTA-UBI_29-41_, showing rapid blood clearance and urinary excretion. In the group of infected animals, the results showed that there was greater accumulation of the radiopharmaceutical at 60 min post-administration, indicating that this is the best time to acquire images [2].

The target-to-non-target ratio (T/NT) of the infected animals was 3.24. Similar values can be found in the literature, 60 min after administration of the product, both for mice (T/NT = 4.62) [36] and for rats (T/NT = 6.10) [34]. On the other hand, studies on [^68^Ga]Ga-NOTA-UBI in BALB/c mice reveal similar ratios: T/NT = 2.60 [31] and 3.24 [41].

The clinical case presented corroborates with the results obtained in the preclinical studies, showing the uptake of the radiopharmaceutical [^68^Ga]Ga-DOTA-UBI_29-41_ at the infectious focus site confirmed via the identification of the pathogen in the surgical bed caused by *Enterobacter cloacae* and *Staphyloccocus aureus* bacteria. This case is part of a pilot project, led by the co-authors of this study, which aims to confirm the presence of bacteria in patients with chronic osteomyelitis using PET/CT images with [^68^Ga]Ga-DOTA-UBI_29-41_. In this study, from the seven cases studied, only one case was negative, because the patient was under antibiotic therapy [42]. These findings are consistent with a study that presented the first PET/CT images with [^68^Ga]Ga-NOTA-UBI_29-41_ in patients. This radiopharmaceutical has been shown to be non-toxic and unlikely to cause adverse effects [43].

It is worth noting that the preclinical studies in this work were conducted only with the MRSA bacterial strain, like previous studies found in the literature [34,35,36,37]. This result indicates that PET/CT using [^68^Ga]Ga-DOTA-UBI_29-41_ is capable of diagnosing *Staphylococcus aureus*, highlighting the importance of planning and carrying out more robust in vitro studies to determine its specificity for different types of bacteria.

Despite the advances in studies with radiolabeled UBI_29-41_, there is still a need to elucidate, through in vitro studies, its specificity in interacting with different bacterial species, as well as to conduct clinical studies on a larger scale, especially with [^68^Ga]Ga-DOTA-UBI_29-41_.

The literature shows that [^68^Ga]Ga-UBI_29-41_ is a selective and specific radiopharmaceutical that can be used to identify bacterial infection; however, clinical studies are in the initial stages. Therefore, it is important to increase the number of patients recruited in future studies in order to obtain greater evidence of accuracy [2].

## 4. Materials and Methods

### 4.1. Synthesis of the [^68^Ga]Ga-DOTA-Ubiquicidin_29-41_

**Figure 9:**
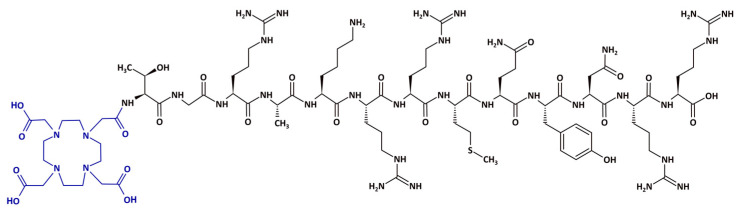
Chemical structure of the DOTA-UBI_29-41_ precursor. Black: UBI_29-41_ peptide. Blue: 1,4,7,10-tetraazacyclododecane-1,4,7,10-tetraacetic acid (DOTA) bifunctional chelator.

The precursor DOTA-Thr-Gly-Arg-Ala-Lys-Arg-Arg-Met-Gln-Tyr-Asn-Arg-Arg-OH (DOTA-Ubiquicidin_29-41_ or DOTA-UBI_29-41_) (Figure 9) and the synthesis cassettes were provided by ABX Advanced Biochemical Compounds (Radeberg, Germany). Initially, the DOTA-UBI_29-41_ molecule was manually radiolabeled with [^68^Ga]GaCl_3_ obtained from a ^68^Ge–^68^Ga GalliaPharm^®^ generator (Eckert & Ziegler, Berlin, Germany) and subsequently implemented in an automated module. [^68^Ga]GaCl_3_ was eluted with 6 mL of 0.1 M HCl and purified in a cationic filter. In the sequence, the [^68^Ga]GaCl_3_ retained in the filter was eluted with 0.5 mL of a solution of 5.5 M NaCl/5 M HCl (97:3) to the reaction vial containing 24.05 nmol of DOTA-UBI_29-41_ diluted in 1.5 mL of 0.1 M NaOAc buffer (pH = 4.5). The solution was heated to 95 °C for 15 min. After the reaction time, the compound was purified in a Sep-Pak C18 cartridge (Waters, Milford, MA, USA), preconditioned with 5 mL of a 1:1 EtOH/H_2_O solution and 5 mL of a 0.9% NaCl solution and eluted with 0.4 mL of a 1:1 EtOH/H_2_O solution. To the final product, 0.9% NaCl solution was added to reach a final volume of 6 mL. RCY and RCP were evaluated before and after the purification process, respectively.

Labeling yield was determined at the end of each synthesis as the ratio between the activity of the radiolabeled product and the activity retained in the module. This last value was obtained as the sum of the activities in the individual components of the module (product vial + empty reaction vial + waste vial + cationic filter + Sep-Pak C18 cartridge + 0.22 μm filter). Activities were measured in a CRC 25R dose calibrator (Capintec, Florham Park, NJ, USA) and the sum was performed without decay correction factor. To test filter integrity, the bubble-point method was automatically carried out at the end of each synthesis. Absence of pyrogens was assessed using an Endosafe^®^-PTS device (Charles River Laboratories, Wilmington, MA, USA) at a dilution factor of 1:50.

Radiolabeling process was standardized and implemented for synthesis using the Modular-Lab PharmTracer automated module (Eckert & Ziegler, Berlin, Germany), before performing preclinical and clinical studies. All solvents and reagents were purchased from Merck KGaA (Darmstadt, Germany) or Sigma-Aldrich Sweden (Stockholm, Sweden), with a high purity grade and were metal-free.

### 4.2. Radiochemical Purity Evaluation

The RCP of [^68^Ga]Ga-DOTA-UBI_29-41_ was evaluated via ascendant TLC, SPE and RP-HPLC.

#### 4.2.1. Ascendant Thin-Layer Chromatography

Ascendant TLC was executed in duplicate, using the system TLC-Silica Gel 60-Aluminium (Merck KGaA Millipore Corporation, Darmstadt, Germany) as the stationary phase and 0.1 M sodium citrate (pH 5.5) as the mobile phase. At the end of the chromatographic development, radioactivity distribution along the stripes was quantified using an AR-2000 radio-thin-layer chromatography imaging scanner (Eckert & Ziegler Radiopharma Inc., Hopkinton, MA, USA), to determine the retention factors and the corresponding activity percentage per peak of [^68^Ga]GaCl_3_ and [^68^Ga]Ga-DOTA-UBI_29-41_.

#### 4.2.2. Solid-Phase Extraction

SPE was performed using a Sep-Pak^®^ C18 cartridge and preconditioned with 5 mL of 1:1 EtOH/H_2_O solution and 5 mL of a 0.9% NaCl solution. [^68^Ga]Ga-DOTA- UBI_29-41_ was eluted from the resin with 0.5 mL of a 1:1 EtOH/H_2_O solution. The activity of the final product vial, waste vial containing [^68^Ga]GaCl_3_ and the C18 cartridge containing the colloidal forms of [^68^Ga]Ga(OH)_x_ were quantified using a dose calibrator. RCP was determined as the ratio of the activity in the product to the total activity (sum of the activities measured in the product vial, waste vial and Sep-Pak).

#### 4.2.3. Reversed-Phase High-Performance Liquid Chromatography

RP-HPLC analysis was performed using Ultra High-Performance Liquid Chromatography (UHPLC) 1290 Infinity II equipment (Agilent Technologies, Santa Clara, CA, USA), coupled to a radiation detector (Eckert & Ziegler Radiopharma Inc., Hopkinton, MA, USA) and the Open Lab ECM^®^ software, version 2.15.26 (Agilent Technologies, CA, USA). A C18 reversed phase analytic column (100 Å, 150 × 4.6 mm, 5 μm, Phenomenex Kinetex^®^), maintained at 25 °C, was used at a flow rate of 1.0 mL.min^−1^, with the following solvent systems: (A) deionized water with 0.1% trifluoracetic acid (TFA) and (B) acetonitrile (ACN) with 0.1% TFA. The gradients used for the mobile phase B were 5–10% (0–2 min), 10–60% (2–12 min), and 60–5% (12–14 min). Signals were recorded as absorbance under UV light (λ = 284 nm) and radioactivity. Retention times (R_t_) for the unlabeled precursor UBI_29-41_, [^68^Ga]GaCl_3_ and the radiopharmaceutical [^68^Ga]Ga-DOTA-UBI_29-41_, as well as the percentages of the activity in each peak, were determined.

### 4.3. Partition Coefficient Determination

The n-octanol/water partition coefficient (P) of [^68^Ga]Ga-DOTA-UBI_29-41_ was determined using the shake-flask method described elsewhere [44]. A 50 µL aliquot (~2 MBq) of the final product was added to a mixture of n-octanol/water (1:1). The tubes containing the mixture were shaken and centrifugated at 5000× *g* for 5 min in a Minispin^®^ plus centrifuge (Eppendorf AG, Hamburg, Germany). Then, they were let to rest until complete separation of the phases occurred. After that, samples of 100 μL were taken from each phase. Radioactivity in the aliquots was quantified in an automated Wizard^2^™ 3” 2480 gamma counter (PerkinElmer, Shelton, CT, USA). From there, the logarithm of P was determined from the ratio of the activity in organic phase to that in the aqueous phase.

### 4.4. In Vitro Studies

#### 4.4.1. Stability of [^68^Ga]Ga-DOTA-UBI_29-41_

Stability of [^68^Ga]Ga-DOTA-UBI_29-41_ was evaluated in saline and in rodent serum. In saline, the radiopharmaceutical was maintained at room temperature and the RCP was determined via ascendant TLC and RP-HPLC at 1, 2 and 3 h after labeling, following the protocols described above. In rodent serum, 50 μL (~2 MBq) of the radiopharmaceutical was incubated in 450 μL of serum at 37 °C and under gentle shaking at 450× *g.* At 0.5, 1 and 2 h, aliquots of the mixture were removed and evaluated via ascendant TLC, as previously described.

#### 4.4.2. Serum Protein Binding (SPB)

SPB was determined via incubation of 50 μL of [^68^Ga]Ga-DOTA-UBI_29-41_ with 450 μL of rodent serum at 37 °C and under gentle shaking at 450× *g*. At 30 and 60 min, proteins were separated via ultracentrifugation at 14,000× *g* using Amicon^®^ 10 kDa filters (Merck Millipore, São Paulo, Brazil). The supernatant and pellet were conditioned in microtubes and their radioactivity was quantified in an automated gamma counter. The percentage of SPB was defined by the ratio between the radioactivity in the pellet and the total radioactivity, given by the sum of the radioactivities of the pellet and the supernatant.

#### 4.4.3. Production of the Methicillin-Resistant *Staphylococcus aureus* Bacteria

Iberian Clone MRSA was obtained from the Unité des Agents Antibactériens—Institut Pasteur, and the BEC/BMB9393 (Brazilian Epidemic Clone) MRSA strain was obtained from the Instituto de Microbiologia Paulo de Goés—Universidade Federal do Rio de Janeiro. Both clones were epidemic MRSA strains with different genetic backgrounds and were characterized by pulsed-field gel electrophoresis (PFGE) and multi-locus sequencing type (MLST) typing (BEC ST239, Iberian MRSA ST247) [45].

The MRSA bacterial strain was prepared in two steps: pre-inoculum and inoculum. In the pre-inoculum phase, 10 mL of Luria Bertani (LB) medium and 20 μL of ampicillin (500 mg/mL) were added to MRSA (50–100 IU). The mixture was maintained overnight at 37 °C and under shaking at 200× *g*. In the inoculum phase, 20 mL of LB medium, 500 μL of the pre-inoculum preparation content and 40 μL of ampicillin (500 mg/mL) were combined. This new product was maintained at 37 °C and under shaking at 200× *g* until it reached an absorbance of 0.8–1.0. This quantification was carried out with an LGI-VS-721N spectrophotometer (LGI Scientific, São Paulo, Brazil). At this point, the content was manually homogenized and 1 mL samples were separated for centrifugation at 14,000× *g* for 5 min. Supernatants were removed and pellets were resuspended in 100 μL of PBS containing 10^9^ CFU.

### 4.5. In Vivo Studies

#### 4.5.1. Animals

Male BALB/c mice aged between 6 and 8 weeks were used in this work. Animals were provided from the vivarium of the Centro de Experimentação e Treinamento em Cirurgia (CETEC) of the Hospital Israelita Albert Einstein (Sao Paulo, SP, Brazil), an Association for Assessment and Accreditation of Laboratory Animal Care International (AAALAC) certified facility. Animals were maintained in conditions free of specific pathogens (SPF), with access to food and water ad libitum, under controlled temperature (22 ± 3 °C) and air humidity (55 ± 10%), in ventilated racks with filtered air and submitted to a light/dark cycle of 12/12 h (lights turned on at 07:00). Paper for nesting and cardboard rolls were available in each animal box for environmental enrichment.

In vivo studies were conducted in accordance with the guidelines established by the Conselho Nacional de Controle de Experimentação Animal (CONCEA), in compliance with the National Institutes of Health Guide for the Care and Use of Laboratory Animals (8th edition, 2011), ARRIVE and under the authorization of the Institutional Animal Care and Use Committee (protocol number 4693/21).

#### 4.5.2. Infection Model

The infection process was induced via the inoculation of 100 μL of the MRSA suspension (10^9^ CFU) into the muscle of the right thigh of the mice. Animals in the control group were injected with 100 μL of 0.9% NaCl into the muscle of the right thigh. Ex vivo biodistribution and in vivo imaging studies were performed 24 h after induction of the infectious model.

#### 4.5.3. Ex Vivo Biodistribution Study

For the ex vivo biodistribution study, [^68^Ga]Ga-DOTA-UBI_29-41_ was administered intravenously through the caudal vein of the mice (~5 MBq/100 μL) in control and infected animals. At 30 and 60 min after administration, animals were euthanized via anesthetic overdose (combination of 300 mg/kg ketamine and 30 mg/kg xylazine) administered intraperitoneally. Organs were removed and weighed, and the radioactivity was quantified in a gamma counter. Percentages of injected activity per gram of organ (% IA/g) were determined, as well as the ratio of target (right tight muscle) to non-target (left tight muscle).

#### 4.5.4. Preclinical PET/CT Imaging

For this study, 5.2 MBq of [^68^Ga]Ga-DOTA-UBI_29-41_ was administered intravenously through the caudal vein of the mice with infectious process. Images were obtained using a microPET/SPECT/CT Albira instrument (Bruker Biospin Corporation, Woodbridge, CT, USA) with the animals under anesthesia with isoflurane at 2% in O_2_. The images were registered in dynamic mode with 90 frames, for 1 h of acquisition beginning at the administration time. Computed Tomography anatomic images were obtained after PET recordings: a field of view of 80 mm, 35 kV, 400 μA and 400 projections. PET/CT images were reconstructed using the Albira software, version 5.6 (Bruker Biospin Corporation, CT, USA) and processed and evaluated using PMOD software, version 3.1 (PMOD Technologies, Zurick, Switzerland).

#### 4.5.5. Clinical Case

The clinical case involves a 39-year-old male patient who underwent multiple operations because of a comminuted fracture in the right tibia. The treatment was complicated by chronic osteomyelitis, and a surgical cleaning of the infected area and placement of bone cement was carried out in 2022. In January 2023, a biopsy of the affected area revealed the presence of *Enterobacter cloacae*, and the patient was treated with antibiotics. One month after the end of the treatment with antibiotics, signs of infection persisted. For this reason, the patient was included in the clinical study approved by the Research Ethics Committee, under CAAE 47052521.9.0000.0071, to conduct a PET/CT scan with [^68^Ga]Ga-DOTA-UBI_29-41_ for evaluation of persistent osteomyelitis in the right tibial plateau.

Whole-body PET/CT images were acquired on a Biograph mCT 40 (Siemens Healthineers, Erlangen, Germany) 60 min after the intravenous administration of 185 MBq of [^68^Ga]Ga-DOTA-UBI_29-41_. Anatomical tomographic images were obtained using the CARE Dose4D dose modulator, following the parameters 100 kV; slice 1.5 mm; pitch 1.3; rotation time 0.5 s. In the sequence, metabolic PET images were acquired with a 200 × 200 matrix; zoom 1, 2 min per bed position, and reconstructed with the UltraHD-PET method employing 2 iterations, 21 subsets and a 3 mm Gaussian filter.

### 4.6. Statistical Analysis

The data obtained underwent statistical analysis using GraphPad Prism^®^ software, version 8.3.1(332) (GraphPad Software, San Diego, CA, USA). Variables were presented as “mean ± standard deviation (SD)”. Student’s *t*-test was used to compare two means, while for comparisons involving three or more groups, Analysis of Variance (ANOVA) followed by Tukey’s multiple comparisons test was applied. Differences between means were considered statistically significant at *p* ≤ 0.05.

## 5. Conclusions

Synthesis of the radiopharmaceutical [^68^Ga]Ga-DOTA-UBI_29-41_ was standardized and implemented in the routine of a hospital radiopharmacy through in vitro and in vivo studies. The final product presented all the features required for a radiopharmaceutical to be used in intravenous administration, as well as a specificity for the infectious processes caused by *Staphylococcus aureus* bacteria.

## Figures and Tables

**Table 1:** Stability of [^68^Ga]Ga-DOTA-UBI_29-41_ in saline after module-based radiolabeling (*n* = 6).

Time (h)	Module-Based Radiolabeling RCP (%)	
	Ascendant TLC	RP-HPLC
0	99.78 ± 0.06	99.63 ± 0.17
1	99.81 ± 0.04	99.64 ± 0.14
2	99.75 ± 0.08	99.73 ± 0.25
3	99.80 ± 0.06	99.81 ± 0.16

## Data Availability

The data presented in this study are available on request from the corresponding author. The data are not publicly available due to privacy.
